# Auditory and Vestibular Involvement in Congenital Cytomegalovirus Infection

**DOI:** 10.3390/pathogens13111019

**Published:** 2024-11-20

**Authors:** Swetha G. Pinninti, William J. Britt, Suresh B. Boppana

**Affiliations:** 1 Department of Pediatrics, University of Alabama at Birmingham, Birmingham, AL 35233, USA; spinninti@uabmc.edu (S.G.P.); wbritt@uabmc.edu (W.J.B.); 2 Department of Microbiology, University of Alabama at Birmingham, Birmingham, AL 35233, USA; 3 Department of Neurobiology, University of Alabama at Birmingham, Birmingham, AL 35233, USA

**Keywords:** cytomegalovirus, congenital infection, hearing loss, vestibular impairment, inner ear inflammation

## Abstract

Congenital cytomegalovirus infection (cCMV) is a frequent cause of non-hereditary sensorineural hearing loss (SNHL) and developmental disabilities. The contribution of cCMV to childhood hearing loss has been estimated to be about 25% of all hearing loss in children at 4 years of age. Although the vestibular insufficiency (VI) in cCMV has not been well-characterized and therefore, underestimated, recent studies suggest that VI is also frequent in children with cCMV and can lead to adverse neurodevelopmental outcomes. The pathogenesis of SNHL and VI in children with cCMV has been thought to be from direct viral cytopathic effects as well as local inflammatory responses playing a role. Hearing loss in cCMV can be of varying degrees of severity, unilateral or bilateral, present at birth or develop later (late-onset), and can progress or fluctuate in early childhood. Therefore, newborn hearing screening fails to identify a significant number of children with CMV-related SNHL. Although the natural history of cCMV-associated VI has not been well characterized, recent data suggests that it is likely that VI also varies considerably with respect to the laterality, timing of onset, degree of the deficit, and continued deterioration during early childhood. This article summarizes the current understanding of the natural history and pathogenesis of auditory and vestibular disorders in children with cCMV.

## 1. Introduction

Congenital cytomegalovirus (cCMV) infection is the most frequent congenital infection worldwide with a birth prevalence in the US reported to be around 0.64% [1,2]. Cytomegaloviruses belong to the beta-herpesvirus family and have been documented in all populations studied. CMV is the largest virus infecting humans with respect to genome size [3,4]. It was shown that about 21% of congenital hearing loss and about 25% of all hearing loss at 4 years of age is due to cCMV [5,6,7]. In addition, cCMV is a significant cause of neurodevelopmental deficits [8]. In contrast to the well documented natural history of sensorineural hearing loss (SNHL) in children with cCMV, the involvement of the vestibular system and the resulting functional deficits have not been defined. The ‘vestibular system’ is complex and includes peripheral and central vestibular complexes. The peripheral vestibular system is comprised of structures in each inner ear: (1) three semicircular canals (SCC) to assess angular acceleration and (2) two otolith organs (utricle and saccule) to assess linear or gravitational acceleration. The central vestibular complex includes the spinal cord, cerebellum, and ocular-motor cranial nerve nuclei that receive visual, somatosensory, and vestibular input through vestibulo-spinal tracts and outgoing cortical connections. An intact vestibular system is required for two primary functions: gaze stability, which is the ability to maintain vision during head movement that requires vestibular, oculomotor, and visual input and postural control, which is the ability to maintain balance during static and dynamic movements that require input from vestibular, visual, somatosensory, and musculoskeletal systems. The vestibular ocular reflex (VOR) develops rapidly and reaches adult-level function by four years of age.

## 2. Epidemiology

cCMV is the leading nongenetic cause of sensorineural hearing loss (SNHL) [2,6,7,9]. The association between cCMV and SNHL was first described by Medearis in 1964 who observed that 2/5 surviving children with severe cCMV disease called cytomegalic inclusion disease (CID) were hearing impaired [10]. Since CMV is transmitted from having close contact with body fluids, infected children, especially infants and toddlers, consist of a major source of infection in the population including women of child bearing age [7,11,12,13]. Although most (85–90%) CMV-infected newborns do not exhibit abnormal clinical findings at birth (asymptomatic cCMV), between 10% and 15% of these children with asymptomatic cCMV have SNHL at birth or later during early childhood. Cognitive, motor, hearing, and visual deficits occur in about 40–60% of children with clinical abnormalities at birth (symptomatic cCMV). Infected children born to mothers with primary CMV infection during the first trimester pregnancy experience higher rates of SNHL and other neurologic complications than those born to mothers who acquired CMV infection later in gestation [2,14,15,16].

Vestibular insufficiency (VI), also referred to as vestibular dysfunction or hypofunction, results from lesions either in the central or peripheral vestibular systems. VI in children can present as difficulties with postural control, gait instability, and difficulties with gaze stability during head movement, which often leads to delays in gross and fine motor development (postural instability, poor hand-eye coordination, ‘clumsiness’), cognition (poor concentration and ability to multi-task), and in emotional and social development (poor cognitive skills, anxiety). School performance is often affected in children with VI affecting sports participation and reading and writing skills [17,18,19].

In contrast to the natural history of cCMV-associated SNHL, which is well-defined and characterized as late-onset (5 to 50%), progressive (11 to 50%), and/or fluctuating (16 to 23%) [20], VI in children with cCMV with or without SNHL has not been well-documented, despite common embryological origins and anatomical proximity of the inner ear structures. However, cCMV-associated VI is increasingly being recognized as a significant adverse outcome with variable incidence rates (19–92%) [21,22,23,24,25,26,27,28,29,30]. While VI has been reported more frequently in children with symptomatic cCMV and SNHL, it has also been observed in children with asymptomatic cCMV with or without hearing loss [30,31,32,33]. Similar to cCMV-associated SNHL, the nature of VI in these children also appears to be variable with respect to the onset (VI present at the initial assessment vs. detected later), laterality (unilateral vs. bilateral), and severity (mild to severe/areflexia) [27,28,29,30].

## 3. Vertical Transmission

An interesting finding in the epidemiology of cCMV is that higher rates of cCMV are observed in populations with higher seroprevalence rates. This is unlike other congenital infections including rubella and toxoplasmosis. Transplacental CMV transmission can occur following both primary (first time CMV infection during pregnancy) or non-primary infections (CMV seroimmune women before pregnancy). This could be secondary to reactivation of a latent virus or infection with a new CMV strain (reinfection). Although women with primary CMV infection during pregnancy transmit the virus at much higher rates, non-primary maternal infections account for the majority of infected infants [7,31]. In populations with lower CMV seroprevalence in women of child-bearing age, at least half of all infants with cCMV are born to mothers who were CMV seropositive prior to pregnancy (non-primary maternal CMV infection) [31]. Studies from Brazil, India, South Africa and Nigeria where the CMV seroprevalence rates are >95% have shown higher rates of cCMV. Therefore, most cCMV infections in these populations follow non-primary maternal infection [1,8,32,33,34,35].

The frequency of cCMV-associated SNHL was shown to be similar in children born to mothers with primary CMV infection during pregnancy and those born following non-primary infections [36,37,38,39]. However, infected children born to mothers with primary maternal infection may experience severe to profound bilateral SNHL more frequently [40]. The frequency of VI has not been systematically studied in children with cCMV born to mothers with primary and non-primary CMV infection during pregnancy.

## 4. Pathogenesis of CMV-Induced Auditory and Vestibular Disorders

Findings derived from studies of human tissue: Several factors continue to be significant hurdles for studies of inner ear damage and the resulting sequelae in auditory and vestibular function associated with cCMV. These include (1) accurate ascertainment of the timing of fetal infection with CMV, (2) quantitation of viral loads in fetal infection, particularly viral loads in target organs such as the inner ear, (3) individual heterogeneity in the development of host antiviral responses to fetal infection, and (4) perhaps most importantly, the limited availability of post mortem tissue from the inner ear following fetal CMV infection and/or infants with cCMV. This latter hurdle is also compounded by the need to process temporal bone specimens rapidly and specifically to prevent autolysis leading to the loss of inner ear architecture and the cell populations for an accurate interpretation of inner ear histopathology. Even when the timing of maternal CMV primary infection is known, fetal infection can only be reliably identified by amniocentesis only after about 16–18 weeks of gestation [41,42,43]. Therefore, the timing of fetal infection is usually assigned more broadly based on the trimester of maternal infection to early, mid or late gestation. From the available data, maternal infections occurring earlier in gestation such as those in the first to mid-late second trimester are more likely to result in auditory and vestibular deficits in infected offspring than those occurring later during the third trimester [44,45,46]. Together, these findings strongly argue that fetal infection during early auditory development and prior to the onset of hearing are most likely to lead to auditory and, potentially, vestibular damage. These observations are consistent with the lack of well-documented cases of hearing loss or vestibular dysfunction in infants infected as newborns. Although it is currently impossible to definitively assign the timing of a fetal infection following non-primary maternal infections, based on findings from women undergoing primary infection during pregnancy, hearing loss and vestibular dysfunction in infants following non-primary maternal infection are also likely result from fetal infections in the first to mid-second gestational period.

Despite these limitations, there have been important insights from histopathological descriptions of the inner ears from fetuses and newborns infected in utero. The hearing status of autopsied infants was only known in a small number of cases. The two most widely referenced studies have provided data on six fetuses in one series and twenty-one fetuses in the second series [47,48]. A more recent study detailed findings in temporal bones from three infants with cCMV [49]. More recently developed methodologies for detection of CMV or cells associated with host responses in the inner ear were not available for studies that were carried out prior to 2000. However, these early studies have been summarized, and in most cases, the findings from these earlier studies appear consistent with those from more recent studies [50]. Common to most reports is the preservation of the overall morphology of the cochlea and vestibular apparatus, arguing that fetal CMV infection either occurred after early embryological development of the inner ear or that fetal CMV infection in the early embryonic period failed to disrupt critical developmental processes such as anterior–posterior positioning, cellular positioning or other steps in early organogenesis required for formation of structures within the cochlea and vestibular apparatus. Studies of inner ear development have demonstrated that characteristic morphological features of the cochlea and vestibular apparatus are present by week 10–12, with lengthening of the bony labyrinth of the cochlear and semicircular canals reaching adult length by about week 17–18 [51]. Thus, the available data suggests that most infections occurred after the 1st trimester, which is consistent with the immaturity of the maternal–placental interface at this early time in fetal development, which has little or no exposure of the developing fetus to maternal blood. In fact, some investigators have suggested that human pregnancy can be divided into an early phase with little contact between the placenta and the maternal blood (first trimester) and a late phase where there is widespread contact between the maternal blood and the placenta (second and third trimester) [52]. Finally, studies in timed fetal tissue have described that maturation of the stria vascularis and expression of cochlear genes critical for hearing takes place between weeks 15 and 18, consistent with the onset of hearing sometime after week 20 [53]. These data suggest that the interval between gestational age 12–20 weeks represents a critical time in auditory development in which virus infection of the inner ear could lead to abnormalities in hearing in the congenitally infected child. However, the exact timing and mechanisms of cCMV-associated injury to the auditory and vestibular system leading to SNHL and VI are not known.

Histopathology, immunohistochemistry (IHC), and in situ hybridization studies have infrequently demonstrated CMV infection of the neurosensory epithelium and, in particular, hair cells and supporting cells, whereas infected cells can be readily demonstrated in the stria vascularis and in the cells in scala media, including Reissner’s membrane (Table 1). In some cases, infected cells have been detected in the spiral ganglion and cochlear nerve (Table 1). In contrast, inflammatory infiltrates containing CD8+ T lymphocytes, B lymphocytes, and macrophages can be found in several regions of the cochlea, including the stria vascularis, the spiral ganglion, and in some cases the neurosensory epithelium (Table 1). In most cases, the neurosensory epithelium of the cochlea remained intact with minimal if any cell loss except in cases of in which autolysis could not be excluded (Table 1).

In contrast to reported histopathological findings in the cochlea of infected fetuses and infants, findings in the vestibular system have been more variable. Infected cells can be found within several regions of the vestibular apparatus and associated with loss of type I and II hair cells (Table 2). Interestingly, infected cells could be found in epithelial cells and dark cells in the utricle and base of ampullae, cells in the vestibular apparatus that serve an analogous function in the formation of ion gradients as marginal cells of the stria vascularis of the cochlea [47,48]. Cells of the neurosensory epithelia of the vestibular apparatus were infected but only in a minority of cases (Table 2). Similar to findings in the cochlea, inflammatory cells including CD8+ T lymphocytes and macrophages can be detected in the vestibular apparatus (Table 2).

When viewed together, these studies of the histopathology of the inner ear in fetuses and infants infected in utero suggest that the broad cell and tissue tropism of CMV permits infection of numerous cell types in the inner ear, with the most obvious being those sites in the cochlea and vestibular apparatus that are most highly vascularized. However, except in a limited number of cases in which it could be argued that CMV leads to the loss of the neurosensory epithelium, it is difficult to assign a pathway(s) leading to hearing loss or vestibular dysfunction as a result of a direct viral cytopathic effect. Moreover, the presence of cells of innate and adaptive immune response within the infiltrates suggests an active host response against infection and raises the possibility that immunopathology contributes to the clinical phenotypes of auditory and vestibular dysfunction in children infected in utero without a prerequisite of virus-induced cytopathology. Immunopathology and inflammation could be of particular importance during critical periods of auditory development. This potential mechanism would be consistent with the established role of cochlear inflammation in hearing dysfunction that has been reported in a number of known insults to the inner ear that have also been associated with cochlear inflammation [54,55,56,57,58]. Consistent with immunopathology as a potential mechanism of disease in cCMV, studies of brain sections obtained from fetuses infected in utero have suggested a correlation between the density of infiltrating cells of the adaptive and innate immune system and the severity of histopathological findings [59].

The technical and methodological hurdles of studies of human temporal bone samples coupled with the limited availability of tissue from cases of congenital CMV infection has made definitive studies of the pathogenesis of hearing loss and vestibular dysfunction in infants and children with cCMV nearly impossible. As a result, several animal model systems have been developed including murine and guinea pig models. Non-human primate models have been developed but have not been used to systematically study the pathogenesis of inner ear disorders following fetal infection. Although considerable effort has been directed towards the development of informative small animal models of CMV-associated SNHL, a similar interest in models of vestibular dysfunction has yet to materialize. Thus, the following discussion of animal models of inner ear damage following intrauterine CMV infection will be limited to a description of small animal models of hearing loss.

Studies in the guinea pig model of cCMV were initially pioneered by Hsiung and other investigators in the late 1970s [60,61,62]. These investigators demonstrated that pregnant guinea pigs inoculated with guinea pig CMV could transmit the virus across the placenta to the developing guinea pig because the hemochorial structure of the guinea pig placenta permits direct contact between maternal blood and fetal membranes, similar to the human placenta. Subsequent studies have exploited this model to study several aspects of intrauterine transmission of guinea pig CMV, perhaps most extensively in the investigation of immune correlates of protection from intrauterine transmission and disease and for the design and testing of various strategies for immunoprophylaxis, including vaccines. In an early study, Woolf et al. reported intrauterine transmission of gpCMV after maternal intracardiac injection in about 30% pregnant guinea pigs, with similar rates of intrauterine transmission being observed following either 1st or 2nd trimester inoculations [63]. Of the guinea pigs born to infected dams, about 25% had evidence of SNHL, and the rates of hearing loss were similar in offspring infected following 1st or 2nd trimester maternal inoculations [63]. Histopathology of infected guinea pig inner ears revealed the presence of gpCMV in the spiral ganglion of the cochlear, the vestibular ganglion, and endothelial cells of the cochlear modiolar vein in about 25% of guinea pigs infected as embryos [63]. An inflammatory infiltrate was observed in the cochlea of a minority of infected animals [63]. Subsequently, other authors have used this model with modifications of the route of inoculation and demonstrated hearing loss but definite histopathological findings that would point to mechanisms accounting for SNHL in congenitally infected animals are for the most part lacking [64,65].

In contrast to these studies of transplacental transmission and infection of guinea pig fetuses, there is extensive literature of several different murine models of hearing loss associated with cCMV [65]. Because the structure of the rodent placenta prevents direct exposure of fetal membranes to maternal blood, different routes have been used to establish infection with the murine CMV (MCMV), a virus genetically that is closely related to CMV and which shares a similar replication program. These routes have included (1) direct intracranial inoculation of murine embryos and newborn mice, (2) direct inoculation of the placenta, and (3) intraperitoneal inoculation of newborn mice. Although these approaches have contributed to current understanding of the mechanism(s) of SNHL, intracranial inoculation of both fetuses and newborn animals bypasses the presumed hematogenous route of virus spread to the inner ear that is thought to occur during human fetal infection. In addition, intracranial inoculation of newborn mice results in significant cochlear pathology within the inner ear with destruction of the neurosensory epithelium and profound SNHL in infected mice [66]. Furthermore, this route of infections leads to significant damage of the vasculature of the stria vascularis resulting in loss of endocochlear potentials and hearing loss [66,67]. In contrast, murine models utilizing peripheral inoculation of newborn mice have relied on viremia and virus spread to the cochlea, presumably through initial infection of cells within the most highly vascularized region of the cochlea, the stria vascularis [68]. Importantly, results from studies of temporal bones from CMV-infected fetuses have consistently shown virus infected cells within the stria vascularis, a finding in agreement with this proposed route of cochlear infection in murine models [47,48]. In murine models which utilize peripheral inoculation of MCMV, virus infected cells can be demonstrated in the stria vascularis and the spiral ganglion but importantly not in the neurosensory epithelium including the supporting cells [69]. Even though a significant number of infected animals develop severe to profound SNHL following peripheral inoculation, the neurosensory epithelium remains intact, at least at the time of auditory testing [69]. This finding is again consistent with findings from autopsy studies of inner ears of fetuses infected with CMV and suggests that disease mechanisms other than direct virus-mediated destruction of the neurosensory epithelium leads to SNHL that follows CMV infection during auditory development [47,48]. In addition to these murine models, investigators have attempted to model early infection via either direct intracranial injection of the developing mouse embryo or via injection of the murine placenta. In a recent study, intracranial inoculation of embryonic day 13 mice resulted in significant structural abnormalities of the developing brain and a high rate of hearing loss in surviving mice [70]. In this study, a titration of the viral inoculum was defined that resulted in a significant number of surviving mice, a major advance when compared to previous studies that utilized this approach and reported very low rates of survival to delivery of infected embryos. However, cochlear histopathology was not reported in mice infected during embryonic development, with and without hearing loss, so histologic correlates of hearing loss are not available [70]. Finally, a novel model of infection of a developing mouse embryo used intraplacental infection of pregnant mice on day 12.5 of gestation and reported that approximately 37% of embryos could be infected, with about 1/3 of embryos being resorbed or lost at that time of delivery [71]. When tested on PNd30 and 70, the group of infected mice had evidence of SNHL as compared to uninfected mice. Histopathological examination of these mice revealed spiral ganglion neuron loss but no other histologic changes in the cochlea [71]. A second group of investigators has confirmed successful transmission of MCMV to the developing brain of the embryo using this same approach [72].

Although several murine models of hearing loss that follow intrauterine infection have been reported, each has technical and/or physiologic limitations that could hinder the translation of data from these systems to the human disease. An important characteristic of auditory development in the mouse is that much of it takes place during the postnatal period, such that auditory development in the newborn mouse parallels that of a mid-to-late 2nd trimester human fetus, including the expression of cochlear genes required for the transduction of the mechanical signal sound into an electrical signal for propagation to the cochlear nuclei and then to the cortex [73,74,75]. The establishment of critical cellular connectivity and generation of ion gradients within the scala media generating the endocochlear potential take place in the postnatal period and result in the onset of hearing in mice between the 2nd and 3rd week of postnatal life. Leveraging this unique feature of murine auditory development, we established a model in which newborn mice (<12 h of age; PNd0) are injected intraperitoneally (ip) with low doses of MCMV [68]. Virus spreads hematogenously, and by PNd4, significant amounts of virus can be detected in the cochlea [47,68]. SNHL developed in approximately 50% of mice and, importantly, recapitulated the hearing loss phenotypes in human infants infected with CMV in utero, including (1) late-onset SNHL, (2) progressive hearing impairment with increasing hearing thresholds over the following three months after initial testing on PNd32, (3) unilateral hearing loss, and (4) fluctuating hearing thresholds over the first three months of postnatal life [6,68,69]. This model has revealed several novel findings, including detection of the virus frequently in cells of the stria vascularis together with activated mononuclear cells, as detected via Iba-1 staining; however, the infection was very focal, while the mononuclear cell infiltration was more generalized and often present in areas of the cochlea without evidence of virus infection [68,69]. In addition, histologic findings of loss of afferent synapses on hair cells reminiscent of the synaptopathy following noise-induced hearing loss was noted [75,76]. Furthermore, loss and disorganization of neurofilaments connecting hair cells and SGN was readily detected [69]. Spiral ganglion neuron (SGN) loss was noted in all regions of the cochlea, and the magnitude of the SGN loss correlated with the degree of hearing loss, as measured via auditory brainstem responses (ABR) [69]. Finally, infection and/or loss of hair cells and supporting cells of the neurosensory epithelium were not observed in infected mice with abnormal ABR and otoacoustic emission (OAE) testing and altered latency of wave 1 of the ABR, both suggestive of hair cell dysfunction [68,69].

Findings from this murine model recapitulated phenotypes and histopathological findings from studies of inner ears from CMV-infected human fetuses, but to date, no single finding has provided a unifying mechanism(s) that could account for the different hearing phenotypes observed in infected mice. However, further examination of the host response to cochlear viral infection suggested the possibility that virus-induced cochlear inflammation could contribute to the loss of hearing in infected mice and potentially could account for different phenotypes of hearing loss. Consistent with this possibility was the correlation between cochlear inflammation and hearing loss [69]. The role of virus-induced inflammation in hearing loss was then directly tested by decreasing cochlear inflammation with corticosteroid treatment, which, importantly, lowered cochlear inflammation without altering viral loads in the cochlea. Treatment of infected mice early, after infection but not at later time points, with anti-inflammatory agents including corticosteroids decreased cochlear inflammation, prevented loss of SGN, and protected mice from hearing loss [69]. Subsequent studies have shown that early in auditory development there is a direct relationship between the quantity of cochlear virus and the degree of cochlear inflammation, which in turn, correlated with hearing loss. Modifying the cochlear viral load early after infection via treatment with antiviral monoclonal antibodies prevented hearing loss, suggesting that some level of cochlear infection and corresponding inflammation can be tolerated during auditory development, but when this level exceeds an as yet undefined level, virus-induced inflammation leads to hearing loss. Again, a specific mechanism that would account for hearing loss resulting from virus-induced inflammation has not been identified, but interestingly, there was a strong correlation between the level of virus-induced inflammation and the dysregulated expression of multiple cochlear genes that have been classified as deafness-related genes (DRG) based on hearing loss phenotypes in humans and engineered mice with mutations or loss of expression of these genes. While it is not possible to assign a specific mechanism of hearing loss to virus-induced inflammation in this model of cCMV-associated hearing loss, the dysregulation of multiple cochlear genes involved in the development of hearing function suggests significant damage to the developing auditory system following cochlear infection. Perhaps more importantly, these results are consistent with findings of inflammatory cell infiltrates and limited evidence of direct viral cytopathology in cochleae from fetuses and infants with cCMV infection, which together suggests that virus-induced inflammation could be the proximal cause of hearing loss that follows intrauterine CMV infection.

## 5. Evaluation and Follow-Up

Outcomes among children with cCMV vary widely. Of the children with symptomatic cCMV, the majority develop permanent complications including hearing loss, motor and cognitive deficits, and visual deficits [8]. SNHL occurs in about 50% of those with symptomatic cCMV and in 10–15% of asymptomatic children [2,6]. The risk factors that have been associated with a higher frequency of SNHL in children with symptomatic cCMV include primary maternal infection during the first trimester, evidence of multi-system involvement at birth, imaging abnormalities at birth, and intrauterine growth restriction. Additional risk factors include exposure to ototoxic drugs, a prolonged neonatal intensive care unit (NICU) stay, or the need for ventilatory support. However, predictors of outcomes in children with asymptomatic cCMV and infected infants born to mothers with non-primary infection have not been defined. Most infants with asymptomatic cCMV are not diagnosed at birth because of the absence of clinical findings.

Audiologic evaluation: All newborns undergo hearing screening using either otoacoustic emission testing (OAE) or an automated auditory brainstem-evoked response (ABR). Those who do not pass newborn hearing screening (NHS) undergo diagnostic ABR to confirm hearing loss. Since ABR testing in older infants may require sedation, it is optimal to complete the diagnostic testing as early as possible. Other protocols such as visual reinforcement audiometry (VRA) can be utilized at seven months of age or older. Pure tone audiometry and other standard protocols in a soundproof environment are used for testing hearing function in older children. In addition, genetic evaluation should be carried out in all children with hearing loss, including CMV-associated SNHL, to determine whether a genetic abnormality plays a role in hearing loss.

Evaluation of vestibular function: Vestibular assessments are not routinely recommended during initial evaluation or follow-up for children with cCMV because of the lack of awareness of the significant impact of VI in children with cCMV, they are labor-intensive and expensive, testing methodologies are not readily available, and there is a shortage of personnel experienced in performing vestibular assessments in young children. In addition, children with VI often present with non-specific symptoms and signs and disorders of balance, and vision in children with symptomatic cCMV are most often attributed to CNS involvement. Given the complexity of the vestibular system, no single assessment allows an evaluation of the entire vestibular system and requires multiple age-appropriate evaluations (Table 3) [3,77]. Otolith organ integrity is assessed by measuring the cervical vestibular-evoked myogenic potential (cVEMP) for saccular function and the ocular vestibular-evoked myogenic potential (oVEMP) for utricular function. The integrity of the lateral SCC and associated brainstem level reflexes is assessed using the vestibulo-ocular reflex (VOR) at varying frequencies (caloric tests, video head impulse test (vHIT), or rotary chair) and the vHIT for all three SCCs. Clinical dynamic visual acuity (cDVA) is used to test the integrity of gaze stability and assess the functional use of the VOR, while postural control and balance is assessed via multiple, standardized gross, fine motor, and balance tests (sensory organization test (SOT) and Bruininks–Oseretsky test of motor proficiency (BOT)) and motor developmental scales (Table 3). The complexity of vestibular system evaluations highlights the urgent need to develop simple, reliable, and practical screening tools to assess the vestibular system.

In children with cCMV, hearing loss can also develop postnatally during early childhood (late-onset). Furthermore, in both symptomatic and asymptomatic cCMV, the hearing deficit can continue to worsen (progressive SNHL) [6,7,78,79]. As predictors or biomarkers of cCMV-associated SNHL are not known, all infected children should undergo a hearing assessment every six months during early childhood (5–6 years of age) and then annually. Detection of hearing loss early during critical speech and language development allows intervention so that outcomes can be improved. In addition to bilateral hearing loss, unilateral loss is also known to lead to speech delay and overall development [80,81,82,83].

As discussed earlier, the occurrence and natural history of VI in children with cCMV have not been studied. A brief discussion of the available data on VI follows. In an early study of vestibular system assessments in cCMV, using hot and cold caloric tests, Pappas reported that 6/11 (54%) of children with asymptomatic cCMV had either a complete lack or hypoactive vestibular responses when vestibular testing was performed using hot and cold caloric tests in 11 children with asymptomatic cCMV [21]. Bernard et al. documented VI in 92% of a cohort of children with cCMV in whom rotary chair testing was performed, but only children with SNHL were included in the study [26]. More recently, in a large retrospective study of 130 children with cCMV who underwent comprehensive audiovestibular evaluations performed at a median age of 21 months, Chebib et al. reported that VI occurred more frequently than SNHL (58% vs. 48%, respectively) [28]. Dhondt et al. carried out comprehensive vestibular evaluations longitudinally beginning at 6 months of age in a cohort of 185 infected children [30]. The mean age at the diagnosis of SNHL was 4.2 m (SD 8.2), compared to 17.3 months (SD 11.3) for VI. Although both SNHL and VI occurred in 31 (16.8%) children, the laterality (uni/bilateral and left ear vs. right ear) and severity of SNHL and VI did not always correlate. This is the only study to undertake comprehensive and longitudinal vestibular assessments in cCMV. However, the findings are limited by the fact the cohort includes predominantly children with symptomatic cCMV. Two recent studies have reported the findings of vestibular assessments in children with cCMV identified on newborn CMV screening. With a cohort of 40 children with asymptomatic cCMV who were identified on newborn screening, Pinninti et al. reported findings of comprehensive vestibular, gaze, and balance assessments (mean age—7.5 years) [27]. While incidence of hearing loss was 17.5%, VI was reported in 45% of the cohort, and saccular abnormalities in 44.7% and SCC abnormalities in 44.8% of the cohort were documented. Additionally, DVA (49%), balance (42%), and the capability to utilize somatosensory, vestibular, and visual inputs for sensory organization were affected in a third to half of the cohort. In another study of 38 children with cCMV identified via universal newborn screening in Finland, Kokkola et al. documented VI more frequently than SNHL (19.4% vs. 10.5%, respectively) [29]. These two studies highlight the finding that VI occurs more frequently than SNHL in children with cCMV, even in children with asymptomatic cCMV with normal hearing.

Late-onset, progressive, and fluctuating hearing loss most often occurs during early childhood, although continues to occur through adolescence among children with asymptomatic cCMV. However, after 5 years of age, the risk of developing hearing loss may not differ from uninfected children [6,7,84]. Compared to uninfected infants, cCMV-infected children fail their NHS at higher rates (1–2% vs. 5–6%) [7]. About 50% of children with CMV-related SNHL will have normal hearing at birth but develop late-onset loss as well as continued worsening of the loss over time [7,78]. The only study with longitudinal vestibular assessments in children with cCMV showed that VI can be late-onset in about a third of infected children and can fluctuate in 23% of children with the deficits, suggesting that VI can also be late-onset, progressive, and fluctuating [30].

Studies have identified motor, balance, and vision abnormalities in children with cCMV but mostly in those with symptomatic infection or in select populations [21,24,27,30,85,86,87,88,89,90,91,92]. However, the data on children with asymptomatic cCMV and in children identified on newborn CMV screening are sparse. Therefore, the epidemiology and natural history of VI in asymptomatic cCMV remains to be defined.

## 6. Management

Antiviral therapy: CMV replication will be inhibited by ganciclovir and valganciclovir, by interfering with viral DNA synthesis [93]. In a randomized trial of infants with severe symptomatic cCMV, including those with central nervous system (CNS) involvement, six weeks of IV ganciclovir demonstrated some benefit in maintaining normal hearing and preventing progression of loss [94]. A follow-up study of children with symptomatic cCMV with or without CNS involvement comparing six weeks of valganciclovir with six months of treatment showed that hearing and neurological outcomes were not different at six months. However, better outcomes were observed in children who received the six months of treatment at one and two years of age [95]. However, long-term follow-up data on children who participated in the two treatment trials is lacking, and therefore, the long-term benefit of antiviral treatment is unknown. A retrospective study of symptomatic children who received valganciclovir reported a measurable worsening of hearing in many of the children over time, suggesting that valganciclovir might only provide short-term improvement in hearing outcomes [96]. A six-month treatment with valganciclovir is currently recommended for infants with moderate to severe symptomatic cCMV [95,97]. Since the benefit of antiviral treatment in children with asymptomatic and mild symptomatic cCMV has not been studied, antiviral therapy is not recommended for these groups [97]. Similarly, the role of antiviral treatment has not been evaluated in children with VI.

Multidisciplinary approach: Children with cCMV should be monitored closely for early identification of hearing loss and VI, allowing the provision of appropriate interventions by a multidisciplinary team that includes audiologists, otolaryngologists, speech pathologists, physical therapists, and educational specialists. All infected children should undergo an ophthalmologic evaluation. Participation in early intervention services will provide the infected children with individualized services. In children with early hearing loss, interventions including hearing amplification before the age of six months improves language outcome [82].

Early intervention services: Children with cCMV should undergo periodic audiological monitoring at six-month intervals until five years of age, with three-monthly follow-ups when hearing levels are fluctuating. Since young children experience frequent episodes of otitis media, these children can experience combined conductive and sensorineural hearing loss, which may lead to delays in evaluations and necessitate multiple assessments.

Hearing amplification: In-the-ear and in-the-canal hearing aids are appropriate only for hearing loss less than 60 decibels (dB). Digital and programmable hearing aids have better sound quality, increased precision, and improved speech recognition [83,98]. Assistive listening devices and bone conduction hearing devices such as bone-anchored hearing implants (BAI) can be useful tools for children with mixed (sensorineural and conductive) and those with unilateral severe to profound hearing loss [99]. However, these devices are only implanted in children five years of age or older.

Cochlear implantation: Early implantation during critical stages of hearing development provides improved localization of the sound and better speech perception in noisy surroundings, leading to improved outcomes [79,100,101,102,103]. The US Food and Drug Administration has approved cochlear implantation in children as young as 12 months [103]. Children with unilateral or bilateral SNHL ≥ 40 db HL should be provided with hearing amplification, and cochlear implantation should be considered for those with bilateral severe SNHL [103].

Vestibular rehabilitation (VR): The manifestations of VI in children include motor delays, difficulties with postural control, and gaze instability and could be central or peripheral in origin and unilateral or bilateral in extent of involvement. Therefore, a comprehensive vestibular assessment, including evaluations of postural control, gaze stability, and otolith and utricular functions, should be completed prior to initiating VR [104,105]. The usefulness of VR in adults is well-established and can be individualized based on the nature and the extent of VI. Although the effectiveness of VR has not been demonstrated, the core principles of adaptation, habituation, and substitution remain pertinent [104]. Briefly, adaptation refers to long-term changes in the neuronal response to head movements with the primary goal to improve gaze and balance stability, and habituation refers to a reduction of response after repeated exposure with a goal to improve symptoms of dizziness. Substitution refers to training in the use of alternative sensory input to improve gaze stabilization and balance. A major challenge in using these principles in children is age-dependent neurological development, cooperation, and capability to follow through with instructions. In spite of these challenges, two randomized controlled studies have demonstrated that VR improves motor skills and balance in children with VI [106,107]. In two children with SNHL and bilateral VI, Braswell et al. showed improvement in the DVA score and reading acuity after six weeks of gaze stabilization exercises [108]. Individualized VR protocols have also shown to be effective in children post-concussion and with cerebral palsy [109,110,111,112,113,114]. However, the effectiveness of VR has not been systematically evaluated in children with cCMV.

## 7. Prevention

Behavioural and hygiene interventions have been shown to be effective in preventing primary CMV infection in seronegative pregnant women [115]. The efficacy of CMV-specific hyperimmune globulin (HIG) infusions to prevent or reduce intrauterine transmission in patients acquiring primary CMV infection in early gestation has been evaluated. In early controlled but non-randomized studies, it was shown that CMV HIG was effective in reducing the risk of intrauterine transmission in primary maternal infection. However, the two randomized trials did not significantly decrease the risk of intrauterine transmission by the CMV-HIG compared with the placebo [115,116,117]. Antiviral prophylaxis with valacyclovir has decreased CMV transplacental transmission in primary CMV infection during pregnancy [118,119,120].

Vaccine development: The US Institute of Medicine report in 2000 ranked developing a vaccine decrease in cCMV-related disease burden as a priority. Several CMV vaccine candidates are in different stages of development and testing [121]. A phase two trial of a CMV glycoprotein B subunit vaccine adjuvanted with MF59 demonstrated about 50% protection against primary CMV infection in CMV seronegative women [122]. The vaccine efficacy waned over the duration (15 months) of the study. In a randomized trial of seronegative adolescents, the gB/MF59 vaccine did not demonstrate significant protection from primary infection compared to placebo [123]. A vaccine candidate that expresses gB and the pentamer complex using the mRNA platform is being examined in a phase three trial [121,124]. The development of an effective CMV vaccine has to overcome the fact that the majority of cCMV-infected infants with cCMV are born to women with non-primary maternal infections.

About 50% of all infants with cCMV-associated SNHL will be identified on newborn hearing screening [125]. The majority of children with CMV-associated SNHL experience progression of the deficit during early childhood. Of the children with cCMV who pass their NHS, late-onset SNHL during early childhood will be seen in about 5%. As it is not possible to identify children with late-onset and/or progressive SNHL, especially among those with asymptomatic cCMV, it is necessary to monitor all infected children for SNHL closely during early childhood. The goals of confirmation of hearing loss by three months and provision of interventions by six months of age for those with SNHL can only be realized by implementing universal newborn CMV screening in addition to NHS.

## Figures and Tables

**Table 1:** Inner ear histopathology: cochlear findings.

Number of Inner Ears ^a^	Cochlear Findings ^b^				
**Teissier et al., 2011** [51]					
**6 (14–35)**	**Neurosensory** **Epithelium**	**Stria Vascularis**	**Scala Media/Reissner’s** **Membrane**	**Spiral** **Ligament**	**Cochlear Nerve**
Virus detected ^c^	2/6	5/6	3/6		3/6
Inflammatory cells ^d^	3/6	5/6	0/6		4/6
**Gabrielli et al., 2013 [52]**					
**18 (21)**	**Neurosensory** **Epithelium**	**Stria Vascularis**	**Scala Media/Reissner’s** **Membrane**	**Spiral** **Ligament**	**Cochlear Nerve**
Virus detected ^e^	1/17	15/17	7/17	1/17	0/17
Inflammatory cells ^f^	9/17	17/17	11/17	13/17	
**Tsuprun et al., 2022** [53]					
**4**	**Neurosensory** **Epithelium**	**Stria** **Vascularis**	**Scala Media/Reissner’s** **Membrane**	**Spiral** **Ligament**	**Cochlear Nerve**
Virus detected ^g^	0/4	0/4	1/4	N/A	N/A
Inflammatory cells ^g^	N/A	N/A	N/A	N/A	N/A

Number of inner ears examined (gestational ages of fetal specimens). ^a^ Histological findings in different regions of the cochlea; ^b^ HCMV detected via histological findings of cytomegalic cell or via immunohistochemistry and/or in situ hybridization; ^c^ inflammatory cells included CD8^+^ T lymphocytes and macrophages; ^d^ virus detected in 15/17 ears via PCR, and numbers shown in table reflect detection via immunohistochemistry; ^e^ inflammatory cells included CD8^+^ T lymphocytes; ^f^ in this series, 4 inner ears from 11-, 21-, and 84-day-old infants were studied; ^g^ N/A indicates that this analysis was not to be done either because of autolysis or tissues were not analyzed for these parameters.

**Table 2:** Inner ear histopathology: vestibular findings.

Number of Inner Ears ^a^	Vestibular Findings ^b^				
**6 (14–35)**	**Crista Ampullaris**	**Semicircular Canals**	**Saccule**	**Utricle**	**Vestibular Ganglion**
**Teissier et al., 2011** [51]					
Virus detected ^c^	N/A	3/6	4/6	4/6	N/A
Inflammatory cells ^d^	N/A	4/6	5/6	5/6	N/A
**Gabrielli et al., 2013** [52]					
**18 (21)**	**Crista Ampullaris**	**Semicircular Canals**	**Saccule**	**Utricle**	**Vestibular Ganglion**
Virus detected ^e^	3/17	1/17	2/17	5/17	2/17
Inflammatory cells ^f^	11/17	6/17	9/17	11/17	13/17
**Tsuprun et al., 2022** [53]					
**4 (12–43)**	**Crista Ampullaris**	**Semicircular Canals**	**Saccule**	**Utricle**	**Vestibular Ganglion**
Virus detected ^g^	N/A	2/4	N/A	N/A	N/A
Inflammatory cells ^g^	N/A	N/A	N/A	N/A	N/A

Number of inner ear examined (gestational ages of fetal specimens). ^a^ Histological findings in different regions of the cochlea; ^b^ HCMV detected via histological findings of cytomegalic cell or via immunohistochemistry and/or in situ hybridization; ^c^ inflammatory cells included CD8^+^ T lymphocytes and macrophages; ^d^ virus detected in 15/17 ears via PCR, and numbers shown in table reflect detection via immunohistochemistry; ^e^ inflammatory cells included CD8^+^ T lymphocytes; ^f^ in this series, 4 inner ears from 11-, 21-, and 84-day-old infants were studied; ^g^ N/A indicates that this analysis was not done either because of autolysis or tissues were not analyzed for these parameters.

**Table 3:** Tests of vestibular system, gaze, and balance.

Clinical tests of vestibular function (office-based)	Oculomotor assessment (nystagmus, saccades, smooth pursuit, vergence, eye alignment) Optokinetic nystagmus Vestibulo ocular reflex (VOR) cancellation Head impulse test (HIT) Head thrust test (HTT) Post head shaking Nystagmus Emory clinical vestibular chair test, modified (m-ECVCT) Bucket test (subjective visual vertical—SVV) Dynamic visual acuity (DVA) Single-leg stance Tandem standing Modified clinical test of sensory integration on balance (mCTSIB)
Assessments of otolith function	Utricle Ocular vestibular-evoked myogenic potential (oVEMP) Subjective visual vertical (SVV) with rotary chair (RC) Saccule Cervical vestibular-evoked myogenic potential (cVEMP)
Assessment of semicircular canal (SCC) function	Horizontal SCC Sinusoidal harmonic acceleration (SHA) with rotary chair (RC) Video head impulse test (vHIT) Caloric tests—hot and cold Anterior and posterior SCC Video head impulse test (vHIT)
Functional assessment of gaze stability	Dynamic visual acuity (DVA) Computerized DVA (cDVA)
Balance and gait assessment (static and dynamic)	Sensory Motor Peabody developmental motor scales (PDMS) Berg balance scale (Pediatric) Ghent developmental balance test Bruininks–Oseretsky test of motor proficiency (BOTS-3)—balance subtest Pediatric functional reach Dynamic gait index (DGI) Functional gait assessment (FGA)
Assessment of posture control	Sensory organization test (SOT) Dynamic perturbation test (DPT)

## Data Availability

No new data were created or analyzed in this study.
